# Retinol and vitamin A metabolites accumulate through RBP4 and STRA6 changes in a psoriasis murine model

**DOI:** 10.1186/s12986-019-0423-y

**Published:** 2020-01-13

**Authors:** Hai-meng Wang, Chao Wu, Yan-yun Jiang, Wen-ming Wang, Hong-zhong Jin

**Affiliations:** Department of Dermatology, Peking Union Medical College Hospital, Chinese Academy of Medical Sciences, National Clinical Research Center for Skin and Immune Diseases, Beijing, China

**Keywords:** Psoriasis, Mouse, Retinol, RBP4, STRA6

## Abstract

**Background:**

Psoriasis is a common chronic inflammatory skin disease that features the abnormal proliferation of keratinocytes. This proliferation could partly result from disturbances in vitamin A metabolism. Changes in psoriasis patients of the levels of retinol-binding protein 4 (RBP4), a carrier of retinol (vitamin A); transmembrane protein stimulated by retinoic acid 6 (STRA6); and other retinol metabolic molecules have not yet been fully established. Therefore, we investigated vitamin A-related proteins in mice with imiquimod (IMQ)-induced psoriasis.

**Methods:**

Thirty mice were divided into four study groups: two groups underwent IMQ application for 3 or 6 days (groups A and B, respectively), and two groups underwent Vaseline application for 3 or 6 days (groups C and D, respectively). Blood and skin samples from both lesional and non-lesional areas of the mice were analyzed using enzyme-linked immunosorbent assays, hematoxylin and eosin staining, immunochemistry, real-time reverse transcription polymerase chain reaction, and RNA sequencing.

**Results:**

IMQ-treated mice developed erythema, scales, and skin thickening. Compared with the control groups, IMQ-treated groups had the following changes: 1) interleukin (IL)-17A, IL-23, and tumor necrosis factor (TNF)-α levels were raised significantly in both serum and lesional skin (all *p* < 0.001); 2) retinol levels in lesional skin increased slightly (*p* = 0.364), but no change was evident in serum retinol levels; 3) STRA6 was upregulated in both lesional skin (*p* = 0.021) and serum (*p* = 0.034); 4) RBP4 levels were elevated in serum (*p* = 0.042), but exhibited only an increasing trend (*p* = 0.273) in lesional skin; and 5) proteins and enzymes that mediate retinoic acid formation and transformation were upregulated in lesional skin.

**Conclusions:**

As the demand for vitamin A in psoriatic mice increased, retinol underwent relocation from the circulation to target tissues. RBP4, STRA6, and the transformation from retinol to retinoic acid were upregulated, which may be part of the mechanism of psoriasis skin lesion formation. We propose that a positive feedback mechanism was formed that maintained the severity of psoriasis.

## Introduction

Psoriasis is a T-cell-mediated, genetically determined, environmentally influenced, chronic inflammatory skin condition that affects almost 2–3% of the population [1]. Psoriasis is characterized by a thickening of the epidermis and the presence of immune infiltrates throughout the dermis and epidermis. These infiltrates involve typical pro-inflammatory cytokines, including interleukin (IL)-23, IL-17A, and tumor necrosis factor (TNF)-α. Vitamin A, an essential fat-soluble vitamin, plays an important role in skin cell differentiation, immune system function, and gene transcription [2]. Many medicines that contain vitamin A derivatives have been used to treat psoriasis. Patients have responded favorably to both the external and internal application of these medicines [3–6]. However, the mechanisms that cause disturbance of the vitamin A metabolic pathway in psoriasis remain unknown.

Retinol-binding protein 4 (RBP4) is the carrier that mobilizes retinol (vitamin A) from the liver to target tissues. Humans absorb vitamin A from food and store it in liver cells as retinol [7]. When the body’s requirement for vitamin A increases, retinol is excreted from the liver to the circulation. Retinol then combines with RBP4 to form a complex (holo-RBP4). When this complex reaches a target tissue, it interacts with a specific membrane protein called stimulated by retinoic acid 6 (STRA6), and the RBP4 then comes back into the circulation as apo-RBP4. STRA6 is a transmembrane protein with unique structural characteristics that can catalyze bidirectional retinol transportation and sequester retinol into target cells. It also functions as a cytokine receptor that activates and programs several signaling pathways that drive proliferation and differentiation of human skin cells [8, 9]. STRA6, therefore, links circulating RBP4 with intracellular retinoid metabolism [10]. After retinol is transported into target cells, it is metabolized into retinal and retinoic acid (RA), which further activates downstream signaling pathways that regulate skin proliferation. Various proteins are involved in retinol metabolism, including cellular retinol binding protein 1 and 2 (CRBP1 and 2), cellular retinoic acid binding protein 1 and 2 (CRABP1 and 2), lecithin retinol acyltransferase (LRAT), retinal dehydrogenases (RDH), and members of the cytochrome P450 family (CYP2, 3, and 4) [5].

Moreover, patients with moderate to severe forms of psoriasis are at a greater risk of developing comorbidities such as metabolic syndrome and vascular disorders [11, 12]. In a hospital-based cross-sectional study, metabolic syndrome occurred in 14.3% of the psoriasis patients compared with 10.0% of the controls, and this difference was statistically significant [13]. The connection between psoriasis and metabolic syndrome is not currently understood, but adipocytokines may play an important role in both conditions [14]. RBP4 is believed to be a critical adipocytokine, and the interaction between RBP4 and chronic skin and systemic inflammation in psoriasis could be bidirectional. T-helper 1 and 17 (Th1, Th17) lymphocyte activation may be a shared pathway [15].

Based on the above findings, it is clear that RBP4, retinol, STRA6, and other proteins involved in vitamin A metabolic pathways possess regulatory functions related to psoriasis and its complications. However, the ways in which the expression of these molecules is modified both in the circulation and in skin lesions of psoriasis remain unclear. More investigations are needed to explore the mechanisms of these modifications and to give us deeper insight into the pathophysiology of psoriasis.

The aims of the present study were to create a psoriasis mouse model and to use it to detect the levels of RBP4, STRA6, retinol, and other vitamin A-related molecules in the circulation and in skin lesions.

## Materials and methods

### Animal experiment

**Fig. 1:**
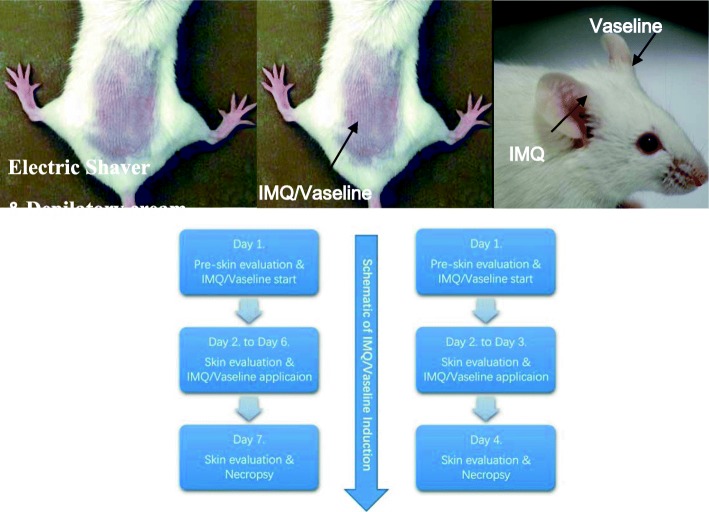
Schematic illustration of animal procedures

Male and female BALB/C mice 8–9 weeks of age obtained from the Charles River Laboratories (Beijing Vital River Laboratory Animal Technology Co., Ltd., China) were used in this experiment. Aldara cream (5% IMQ, 3 M Pharmaceuticals, UK) was used to induce the psoriasis-like lesions and Vaseline cream (Dezhou Chengze Sterilized Production Co. Ltd., Shandong, China) was used as control agents [16, 17]. Mice (*n* = 20) were randomly divided into experimental group A, B (Aldara cream for 3 and 6 consecutive days). Mice (*n* = 10) were also randomly separated into two control groups: group C and D (Vaseline cream for 3, 6 consecutive days) [18–21]. An area about 2 cm × 3 cm in size was shaven on the back of each mouse with electric shaver before the experiment. Depilatory cream (Veet, Reckitt Benckiser, China) was also used to remove the hair. The method described by Van der Fits et al. [16] was used for inducing the psoriatic mouse model. 62.5 mg (contained 3.125 mg of the active compound, IMQ) of Aldara cream topically applied to the back and the right ear. The control groups were treated daily with 62.5 mg white Vaseline cream on the same areas. The day before cream usage was defined as Day 1 and the baselines of modified psoriasis area and severity index (PASI) score was evaluated on this day. After the initial assessment, IMQ or Vaseline cream was used, 24 h after the first application was defined as Day 2. The mice were sacrificed on Day 4 and Day 7 by acute blood loss under anesthesia. Blood and skin from both lesional, non-lesional area and the ears were obtained from each mouse. Schematic illustration for animal procedures are shown in Fig. 1.

### Modified psoriasis area and severity index (PASI) score

The scoring system described by Van der Fits et al. [16] was an alteration of the clinical PASI score. We used a modified PASI score as a tool to evaluate the severity of skin lesion. The indexes included erythema, scales, and thickening. Each index was scored independently on a scale from 0 to 4: 0-none, 1-slight, 2-moderate, 3-marked, 4-maximum, and recorded every 24 h. To help with the evaluation, the skin redness was evalutated with scoring tips introduced by Irfan A. Rather et al. [18], and the thickness of the skin was measured with a micrometer (RF134, Yazhongboke Beijing, China). The total score (erythema plus scaling plus thickening) was used to evaluate the overall severity of skin inflammation (scale 0–12).

### Histopathological analysis

The lesional and the normal skin from group A to D were excised immediately after sacrifice and were fixed in 10% formaldehyde for 48 h. The formalin-fixed skin tissue was then dehydrated in Fully Enclosed Tissue Processor (Leica ASP300S, Leica Biosystems, Germany) and embedded in paraffin. After embedding, the tissue was sectioned vertically at 4 μm, using microtomes (Leica RM2235, Leica Biosystems, Germany). Following this, the section was dried at 72 °C (PHY-III Medical instrument co. LTD., Changzhou, China) for an hour. The staining process was done with autostainer (Leica ST5010, Leica Biosystems, Germany), using Harris’ hematoxylin and eosin for histological analysis.

### Elisa

The blood was drawn from the aorta abdominalis under anesthesia. Blood serum was extracted by centrifuge (D3415R, Bio-One Scientific Instrument Co., Ltd., China) and stored in − 80 °C until use. Skins from both the lesional and non-lesional areas in all the groups were snap-frozen in liquid nitrogen and stored at − 80 °C. Harvested mouse skin tissue (approximately 0.1 g) were prepared in ice-cold buffer containing phosphate-buffered saline (PBS, ZLI-9062, ZSGB-BIO, China) (PH 7.2–7.4) for skin homogenates. The supernatant was collected after centrifugation at 2500 rpm for 20 min at 4 °C. IL-17A (XFM21581), IL-23 (XFM20248), TNF-α (XFM21527), RBP4 (XFM21121), STAR6 (XFM21632) and retinol (XFM21227) levels were detected with ELISA kits which purchased from Shanghai Xinfan Bio-Technology Co. Ltd. (Shanghai, China).

### Immunohistochemistry

Samples were snap-frozen in liquid nitrogen, and stored at − 80 °C until use. The pathologic sections were made using the same protocol as described above. Standard IHC protocol was followed to stain the skin samples using the rabbit monoclonal antibody against RBP4 (ab188230, Abcam), 1:100 diluted with antibody diluent (ZLI-9030, ZSGB-BIO, China). Rabbit polyclonal antibody against STRA6 (H00064220-D01P, Novus), 1:1000 diluted with antibody diluent. Briefly, 4 μm sized paraffin-embedded tissue sections were de-paraffinized with xylene, hydrated through a series of ethanol concentrations. Washed with PBS (ZLI-9062, ZSGB-BIO, China), subjected to antigen retrieval using 10 mM sodium citrate (ZLI-9065, ZSGB-BIO, China) and heated with microwave (EM-GF668, SANYO, Hefei, China). Cooled in room temperature for at least half an hour, washed with PBS. Incubated the sections in 0.5% Triton (Amresco-0694, AMRESCO, USA) for 15 min. Washed thoroughly with PBS. Endogenous peroxidase activity was quenched with 3% H2O2 in methanol for 15 min in a dark moister chamber. Rinsed the sections, then blocked with goat serum (ZLI-9022, ZSGB-BIO, China) for half an hour. Slides were incubated with the respective primary antibody mentioned above and kept in a moist chamber overnight at 4 °C. The next day, washed the slides with PBS several times. Then incubated with secondary antibody kit (PV-9001, ZSGB-BIO, China). After washing, slides were reacted with freshly made DAB (diaminobenzidine) (ZLI-9019, ZSGB-BIO, China) and immediately washed under tap water after color development. Slides were then counter-stained with hematoxylin. Slides were dehydrated through a series of alcohol and mounted with xylene. The slides were observed under a light microscope (DM6000B, Leica Biosystems, Germany). Five high-power fields (×400) were selected at random, specimens were scored according to the intensity of the dye color and the number of positive cells. The intensity of the dye color was graded as 0 (no color), 1 (light yellow), 2 (light brown), or 3 (brown), and the number of positive cells was graded as 0 (<5%), 1 (5–25%), 2 (25–50%), 3 (51–75%), or 4 (>75%). The two grades were added together and specimens were assigned to one of four levels: 0–1 score (−), 2 scores (+), 3–4 scores (++), more than 5 scores (+++).

### RNA isolation and RT-qPCR

RNA extraction was performed with TRIzol (Invitrogen, Carlsbad, CA, USA). RNA (2000 ng) was reverse transcribed using the PrimeScript™ RT reagent Kit (RR037A, TaKaRa, Dalian, China). RBP4, STRA6, and GAPDH mRNA levels were measured by PCR analysis using the SYBR Premix Ex Taq II (RR820A, TaKaRa, Dalian, China). GAPDH was used for normalization. The following primers were used:

GAPDH, forward primer, 5′-TGCACCACCAACTGCTTAG-3′, reverse primer, 5′-GGATGCAGGGATGATGTTC-3′. RBP4, forward primer, 5′-GACAAGGCTCGTTTCTCTGG-3′, reverse primer, 5′-AAAGGAGGCTACACCCCAGT-3′. STRA6, forward primer, 5′-AGCCAAGTCAGACTCCAAGAG-3′, reverse primer, 5′-CAGAGAGCACACTAACTTCTTTCA-3′. All the primers were purchased from Majorbio Bio-pharm Technology Co. Ltd. (Shanghai, China). The relative expression levels of the target proteins were calculated using the 2^−ΔΔCt^ method by normalizing to the reference gene.

### mRNA library construction and RNA-sequencing

RNase H was used to remove the rRNA, DNase I was used to digesting double-stranded and single-stranded DNA in total RNA. Purified RNA from previous steps was fragmented into small pieces with fragment buffer at an appropriate temperature. Then, cDNA was generated in First Strand Master Mix by PCR. The reaction product was purified by magnetic beads, afterward, A-Tailing Mix and RNA Index Adapters were added by incubating to end repair. The cDNA fragments were amplified with adapters by PCR, and the products were purified by Ampure XP Beads. Library was validating on the Agilent Technologies 2100 bioanalyzer for quality control. The double-stranded PCR product from the previous step was heat-denatured and circularized by the splint oligo sequence. The single-strand circle DNA (ssCir DNA) was formatted as the final library, which further amplified with phi29(Thermo Fisher Scientific, MA, USA). DNA nanoball (DNB) which had more than 300 copies of one molecular was made and loaded into the patterned nanoarray and single end 50 bases reads were generated on BGISEQ500 platform (BGI-Shenzhen, China). All the generated raw sequencing reads were filtered to get clean reads stored as FASTQ format. Bowtie2 and HISAT were used to map clean reads to reference gene and genome respectively. Gene expression level (RPKM) is quantified by RSEM. Gene ontology (GO) and pathway annotation and enrichment analyses were based on the GO Database (http://www.geneontology.org/) and Kyoto Encyclopedia of Genes and Genomes (KEGG) pathway database (http://www.genome.jp/kegg/), respectively.

### Statistical analysis

Statistical analyses were performed in SPSS version 20 (IBM, Armonk, NY, USA) and GraphPad Prism version 6 (GraphPad Software, San Diego, CA, USA). Data were given as arithmetical means ± SEM. Statistical analysis was generated with Student’s t-test, Wilcoxon test, Kruskal-Wallis test, and ANOVA. Values of *P* ≤ 0.05 were considered significant.

## Results

### General changes in the skin

**Fig. 2:**
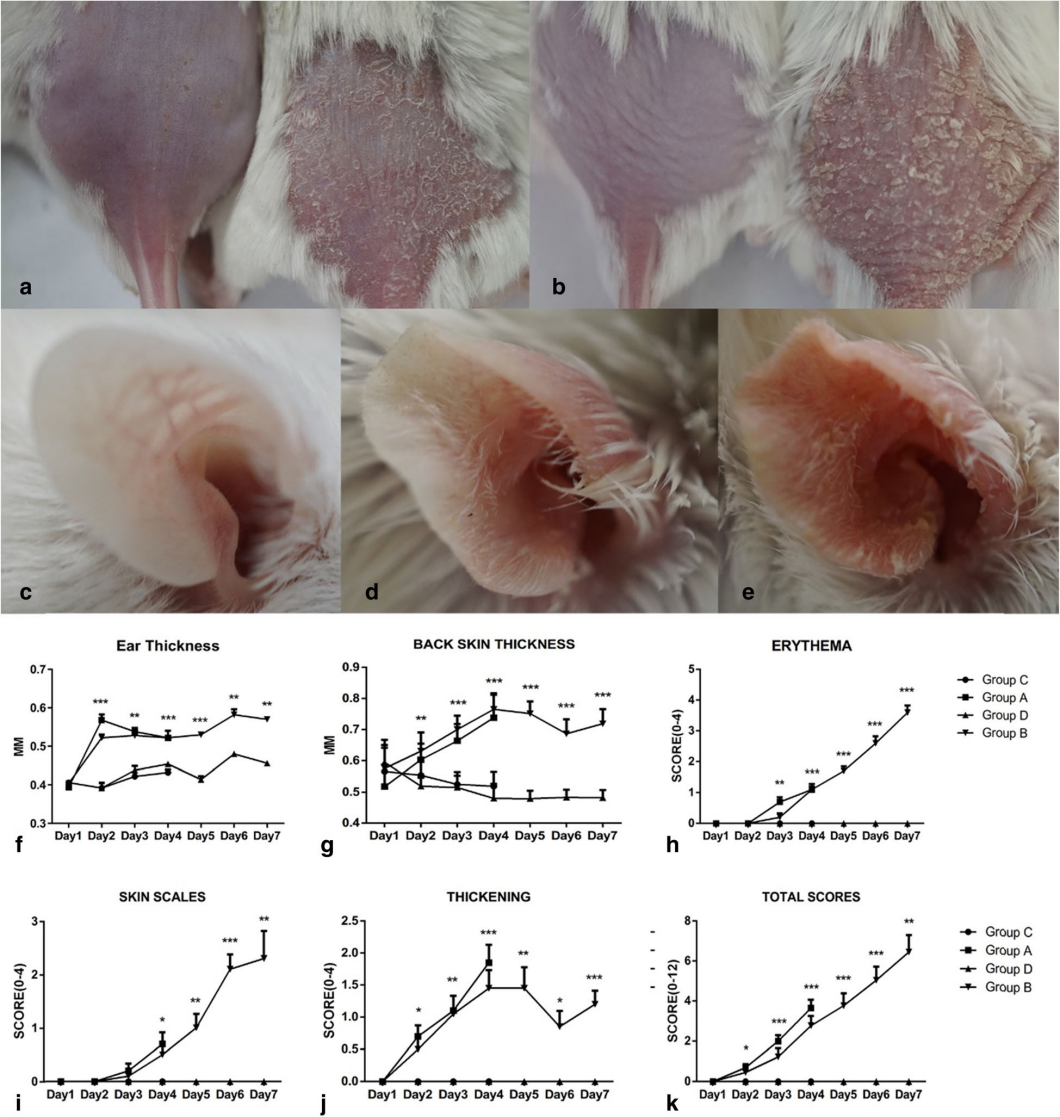
Skin changes in response to IMQ treatment in the study group mice. (**a**) Group A showed skin thickening, erythema and few scales compared with the controls. (**b**) Group B showed profound thickening and erythema, and formation of many sheet-form scales. The psoriasis-like cutaneous changes in IMQ-treated ear also showed a treatment duration-dependent manner, control groups (**c**), group A (**d**) and Group B (**e**). After the IMQ application, the skin thickness increased sharply on the ear (**f**) and back (**g**). Modified PASI scores include erythema (**h**), skin scales (**i**), and thickening (**j**). The total score (**k**) indicating the overall status. *p<0.05; **p<0.01, ***p<0.001

Psoriasis-like lesions with clear erythema and skin thickening were successfully induced in all mice in groups A and B, although the scales were more evident in group B than in group A. Generally, erythema appeared on day 2 and progressed until day 7. Scale formation started on day 3 or day 4 and increased gradually until day 7. Skin thickening increased from day 2 to day 4. The cutaneous changes found in groups A and B are shown in Fig. 2a and b. Few cutaneous alterations were evident after mice were treated with Vaseline. Similar changes were found in the right ears of mice, as shown in Fig. 2c-e. These skin changes became more prominent in group B than in group A. In general, the symptoms accumulated over time. As an independent index of skin inflammation, we measured the thickness of the skin on the backs and ears of the mice (Fig. 2f, g). Based on modified PASI scores, we assessed each parameter daily (Fig. 2h-k). The statistical analysis results are shown in Table 1.

**Table 1:** Statistical analysis of modified PASI scores in the four study groups

*P* values	Day 1	Day 2	Day 3	Day 4	Day 5	Day 6	Day 7
Ear thichkess	/	/	<0.001	0.002	<0.001	<0.001	0.009
Back thickness	/	0.025	0.005	<0.001	0.002	0.023	<0.001
Erythema	/	/	0.003	<0.001	<0.001	<0.001	<0.001
Skin thickening	0.242	0.002	<0.001	<0.001	<0.001	<0.001	<0.001
Scales	/	/	0.542	0.035	0.002	<0.001	0.002
Total scores	/	0.022	<0.001	<0.001	<0.001	<0.001	0.004

In summary, from days 2 to 3 and onward, inflammation was visible and continuously increased in severity in IMQ-treated groups A and B. In contrast, there were no signs of inflammation in Vaseline-treated group C and D.

### Histopathological changes

**Fig. 3:**
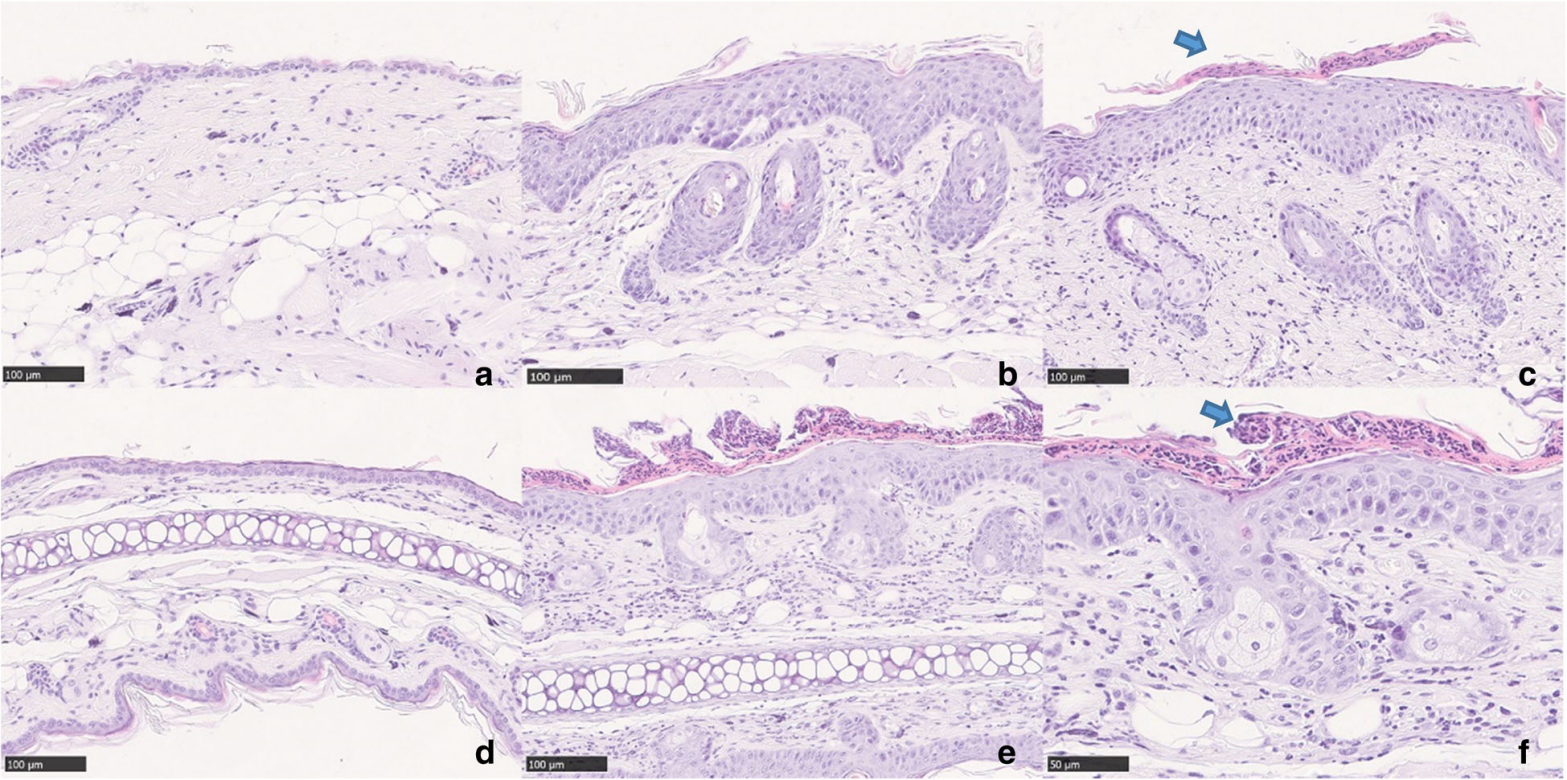
Histopathological changes in IMQ mice. (**a**) Normal skin structure on the back indicated by only 1–2 layers of cells in the epidermis. (**b**) Group A: evident acanthosis, granular layer was absent focally, and moderate infiltration of lymphocytes and histocytes in the dermis. (**c**) Group B: typical Munro abscess was formed (blue arrow). Inflammatory cell infiltration was more severe. Similar changes were observed in the ears of the control groups (**d**) and group B (**e**). Characteristic Munro abscess was indicated with blue arrow in (**f**). **a-f**: hematoxylin and eosin; **a-e** original magnification × 200; F: original magnification × 400

The histopathology of Vaseline-treated skin from control groups revealed a normal epidermal structure consisting of one to two layers of cells only. Normal dermis and subcutaneous structures were found in non-lesional skin (Fig. 3a).

In group A, the histopathological analysis showed severely thickened epidermis made up of five to 10 layers of cells. The granular layer was absent focally, corresponding to areas producing foci of parakeratosis. Severe acanthosis and moderate rete ridge formation could be seen in this group. In the dermis, there were many hair follicles. Moderate inflammatory cell infiltration (mostly lymphocytes and histocytes) was evident in the superficial layer of the dermis (Fig. 3b). The lesional skin on the backs of mice in group B had similarly thickened epidermis, hyperkeratosis accompanied by focal Munro abscess formation, and severe permeation of lymphocytes and histocytes into the superficial and middle level of dermis. The permeation was particularly evident around vessels and collagen fibers (Fig. 3c). Similar changes were also found in the ears of mice (Fig. 3d-f).

### Changes in the levels of pro-inflammatory cytokines and vitamin A-related molecules in the skin

**Fig. 4:**
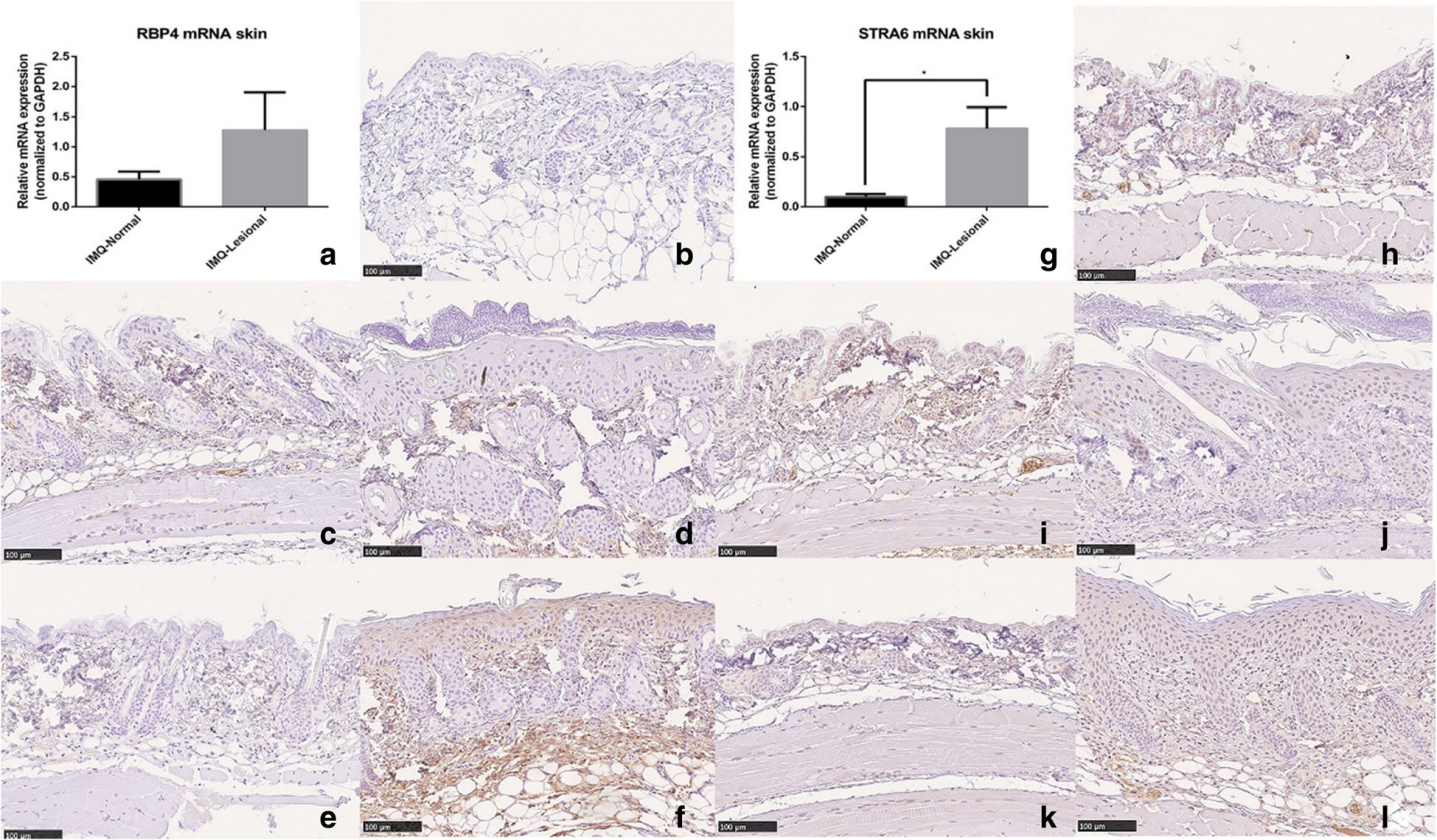
Immunochemistry and RT-qPCR of RBP4 and STRA6 expression. (**a**) Lesional skin had a higher level of RBP4 mRNA expression compared with non-lesional areas (*p* = 0.273). Immunohistochemistry results of RBP4 in the control groups (**b**), normal skin from group A (**c**), lesional skin from group A (**d**), non-lesional skin from group B (**e**), and lesional skin from group B (**f**). Protein levels of RBP4 accumulated in the epidermis over time. (**g**) Compared with normal skin, the STRA6 mRNA level was significantly higher in IMQ lesional skin (*p* = 0.021). STRA6 immunohistochemistry results of the control groups’ skin (**h**), normal skin of group A (**i**), lesional skin of group A (**j**), normal skin of group B (**k**), and lesional skin of group B (**l**). IMQ induced psoriasis-like skin lesion that contained a relatively higher level of STRA6. **b-f** and **h-l**: DAB, original magnification × 200

In normal mouse skin (skin from group C and D), expression levels of RBP4 were low; therefore, we graded the control group as RBP4 negative (Fig. 4b). In contrast, in IMQ-induced skin lesions, RBP4 expression was significantly upregulated in the epidermis. After 3 days of IMQ application, the psoriatic skin in group A (Fig. 4d) showed few changes, with no obvious RBP4 expression in epidermis, although some nonspecific positive dye was presented in the dermis; this expression level was classified as −/±. In group B lesional skin (Fig. 4f), many RBP4-positive cells were observed in the epidermis as well as in the dermis. RBP4 expression was evident mainly in the stratum granulosum and stratum spinosum layers, and in the cytoplasm of keratinocytes. In some areas, there were RBP4-positive cells in the basal layer. In the dermis, the monocyte cytoplasm was strongly dyed and was graded as +/++. In contrast, normal skin from both group A (Fig. 4c) and group B (Fig. 4e) was RBP4 negative. This result was confirmed by RT-qPCR, in which the RBP4 mRNA levels were higher in lesional skin than in non-lesional skin in all IMQ groups, although the difference was not significant (*p* = 0.273; Fig. 4a).

The RBP4 receptor, STRA6, accumulated gradually after IMQ utilization in the lesional skin. In normal skin, STRA6 is expressed in both epidermis and dermis. Because normal epidermis consists of only one or two layers of cells, STRA6 is expressed in all layers of the epidermis, mainly in the cytoplasm. In the dermis, many nonspecific expressions of STRA6 were seen in endothelial cells inside blood vessels (Fig. 4h). Normal skin from mice in group A (Fig. 4i) and group B (Fig. 4k) had similar expression patterns. All three groups were graded as STRA6 +. In contrast, in lesional skin in group A (Fig. 4j), keratinocytes in the stratum spinosum were also dyed light brown, indicating STRA6 expression. However, the overall expression level was similar to that of the control groups, that is, grade +. In lesional skin from mice in group B (Fig. 4l), large numbers of keratinocytes, mainly located in the stratum spinosum and basal layers, expressed STRA6. In the dermis, the number of STRA6-positive cells was also significantly higher than in normal skin; the expression level was designated +~++. In all positive cells, STRA6 staining was present in the cytoplasm and cell membranes. The quantitative results for STRA6 in the lesional and non-lesional groups are depicted in Fig. 4g: STRA6 expression in the IMQ lesional skin was significantly higher than in non-lesional skin (*p* = 0.021).

**Fig. 5:**
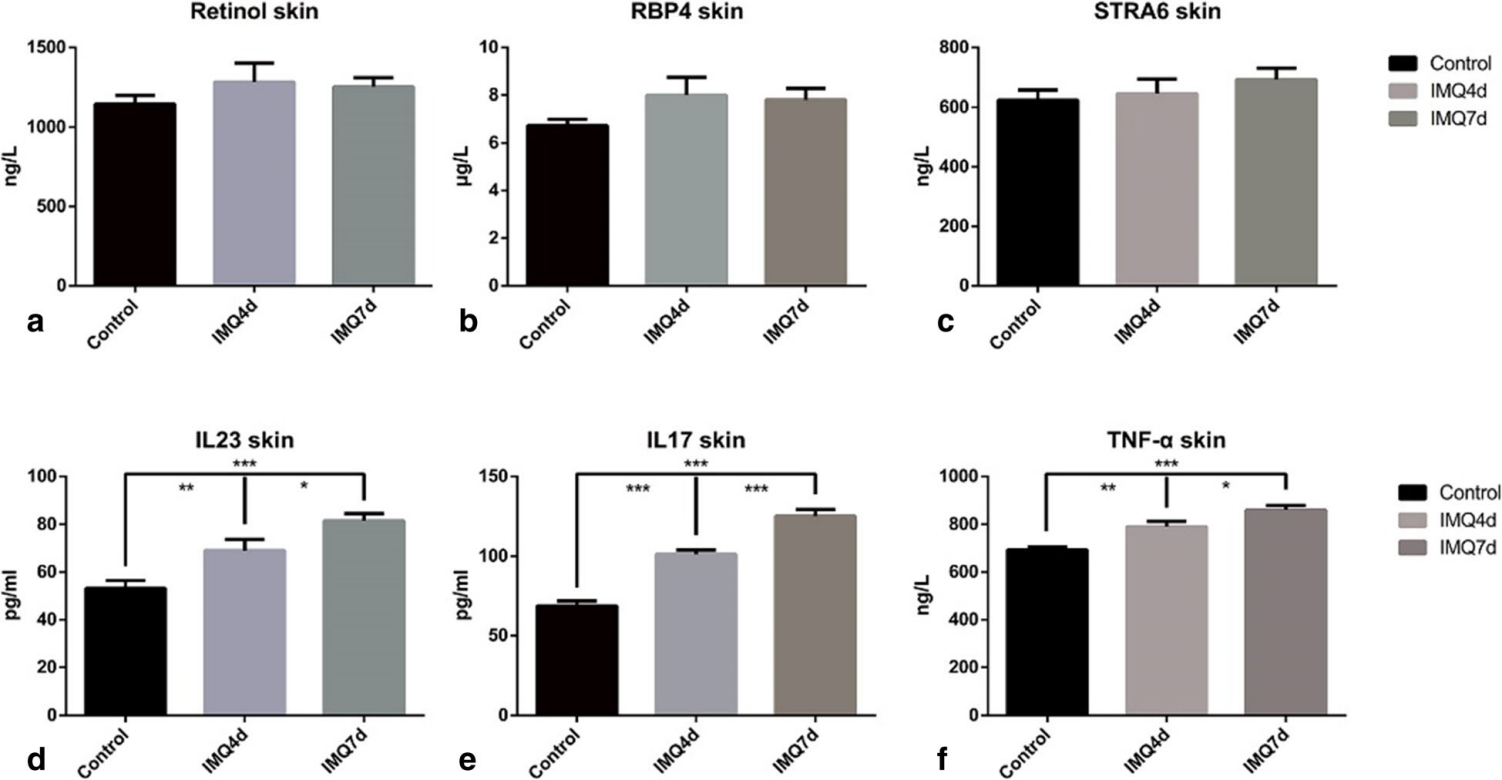
ELISA of vitamin A-related molecules and typical pro-inflammatory cytokines in the skin of the study groups. All results describe comparison with the control groups. (**a**) Retinol levels were slightly higher in lesional skin (*p* = 0.364). (**b**) RBP4 expression was elevated in the IMQ groups (*p* = 0.142). (**c**) STRA6 expression also showed an increasing trend in the IMQ groups (*p* = 0.395). No obvious differences were observed in the two IMQ groups. (**d**) IL-23 increased sharply in the lesional skin of group A (**p = 0.0092) and B (***p < 0.001). Between the two IMQ groups, more IL-23 accumulated in group B (*p = 0.031). (**e**) IL-17A increased in the lesional skin of both IMQ groups (***p < 0.001). Group B had a higher level of IL-17A in lesional skin (***p < 0.001). (**f**) TNF-a expression was highest in group B lesional skin compared with group A (*p = 0.030) and the controls (***p < 0.001)

The RBP4 and STRA6 levels determined using ELISA analysis were higher in IMQ-treated groups than in controls, but the difference was not significant (Fig. 5b, c). Retinol levels in the skin were also detected using ELISA: the IMQ-treated groups showed an increasing trend, but it was not statistically significant (Fig. 5a).

In the current study, we also detected changes in the levels of some typical cytokines in psoriatic lesional and non-lesional skin, which are shown in Fig. 5d-f and Table 2. Levels of pro-inflammatory cytokines, such as IL-17A, IL-23 and TNF-α, were significantly raised in the IMQ-treated groups. The classic IL-17/IL-23 pathway was evidently activated in the IMQ psoriasis mouse model, mimicking the in vivo changes in psoriasis patients.

**Table 2:** ELISA of pro-inflammatory cytokines and vitamin A-related molecules in psoriasis lesional and non-lesional skin

Skin	Controls (*n* = 5)	Group A (*n* = 5)	Group B (*n* = 5)	*P* Value
IL-17A (pg/ml)	78.74 ± 3.03	101.19 ± 2.59	125.22 ± 4.23	<0.001***
IL-23 (pg/ml)	53.15 ± 3.31	69.01 ± 4.73	81.52 ± 3.03	<0.001***
TNF-α (ng/l)	692.65 ± 12.88	790.41 ± 22.52	860.61 ± 18.96	<0.001***
RBP4 (μg/l)	6.73 ± 0.27	8.00 ± 0.75	7.81 ± 0.47	0.142
STRA6 (ng/ml)	623.11 ± 34.22	645.15 ± 48.38	692.66 ± 37.36	0.395
Retinol (ng/l)	1146.17 ± 52.55	1282.22 ± 120.26	1252.41 ± 57.09	0.364

### RNA sequencing

**Fig. 6:**
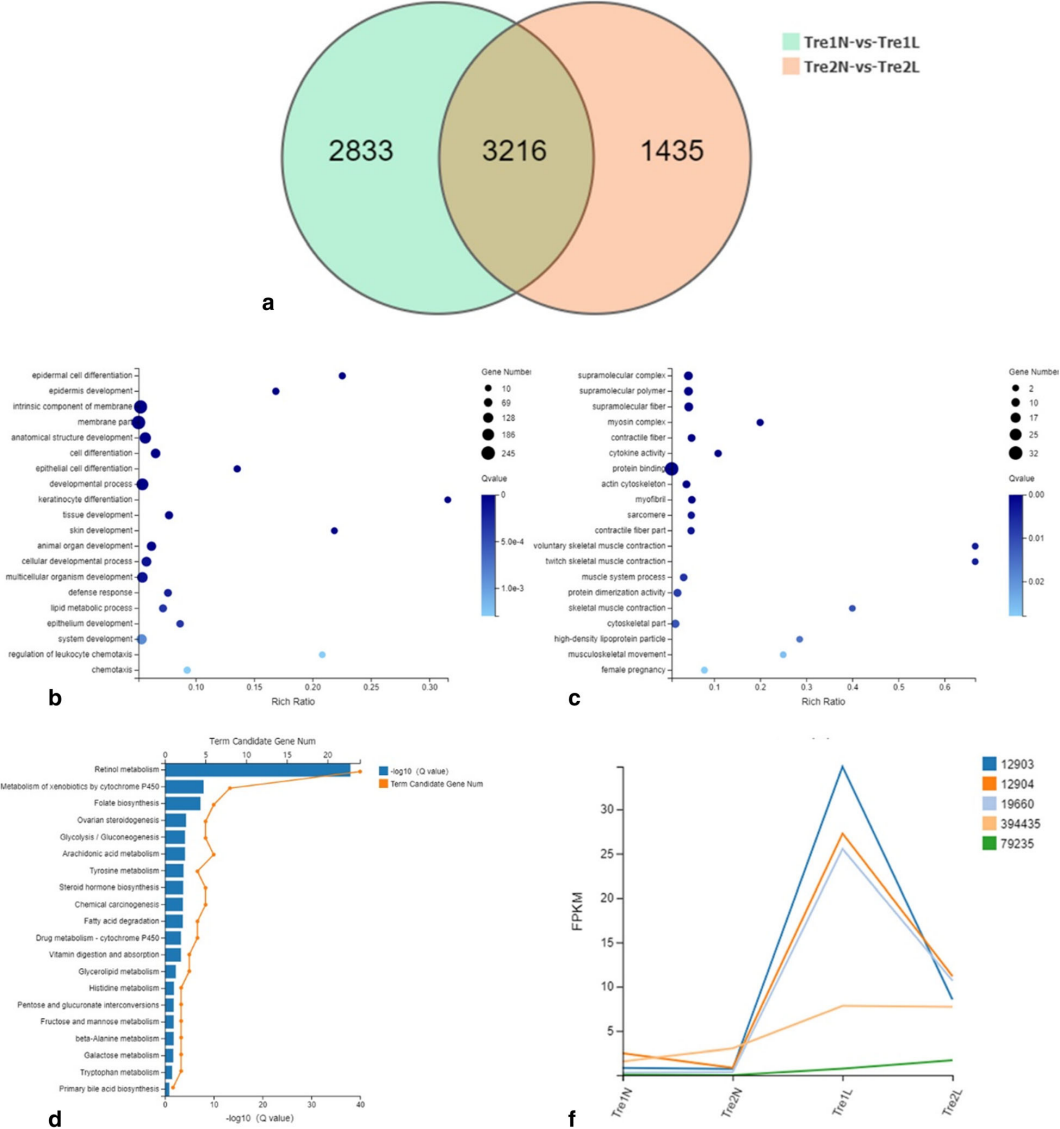
RNA sequencing of lesional and non-lesional skin in the IMQ study groups. **a** Venn diagram showing the number of differentially expressed genes between the different groups. **b**, **c** GO annotation of differentially expressed genes (fold change ≥2) between the lesional and non-lesional skin of groups A and B. Genes with increased expression are depicted in 6B, while genes with reduced expression are in 6C. **d** KEGG pathway enrichment analysis of the genes related to “retinoid metabolic process”. **e** Broken line graph showing the expression of retinol binding protein in IMQ lesional skin compared with non-lesional skin. The serial numbers representing the corresponding gene designations are as follows: 12903, CRABP1; 12,904, CRABP2; 19,660, CRBP2; 394,435, Ugt1a6b; 79,235, lecithin: ROL acyltransferase LRAT. “N” indicates non-lesional skin; “L” indicates lesional skin; “1 and 2” correspond to groups A and B, respectively

Differential gene expression analysis between lesional and non-lesional skin indicated that the expression of 6049 and 4651 known genes changed after IMQ application at 3 and 6 days, respectively. Among these genes, 3216 genes were the same in both groups (Fig. 6a). We then conducted GO annotation analysis for these 3216 genes. Genes that were upregulated in the lesional groups were mainly classified as related to epidermal cell differentiation, epidermis development, keratinocyte differentiation, skin development, lipid metabolic processes, and regulation of leukocyte chemotaxis, among others, as shown in Fig. 6b. These categories matched those involved in the pathophysiologic processes in psoriasis. In contrast, downregulated genes were mainly involved with supramolecular complexes, cytokine activity, and protein binding (Fig. 6c). In GO enrichment analysis, 42 genes were indicated using “retinoid metabolic process” as the search term; among these differentially expressed genes were RBP4 and STRA6. KEGG pathway enrichment analysis of these 42 genes is shown in Fig. 6d.

Using “retinoid binding” as the search term in GO enrichment analysis, five genes were identified: CRBP2, CRABP1, CRABP2, UDP-glucuronosyltransferase 1–6 (Ugt1a6b), and LRAT. In Fig. 6e, we show the expression levels of these five genes in the different treatment groups. All five proteins were more highly expressed in IMQ lesional skin than in non-lesional skin.

**Fig. 7:**
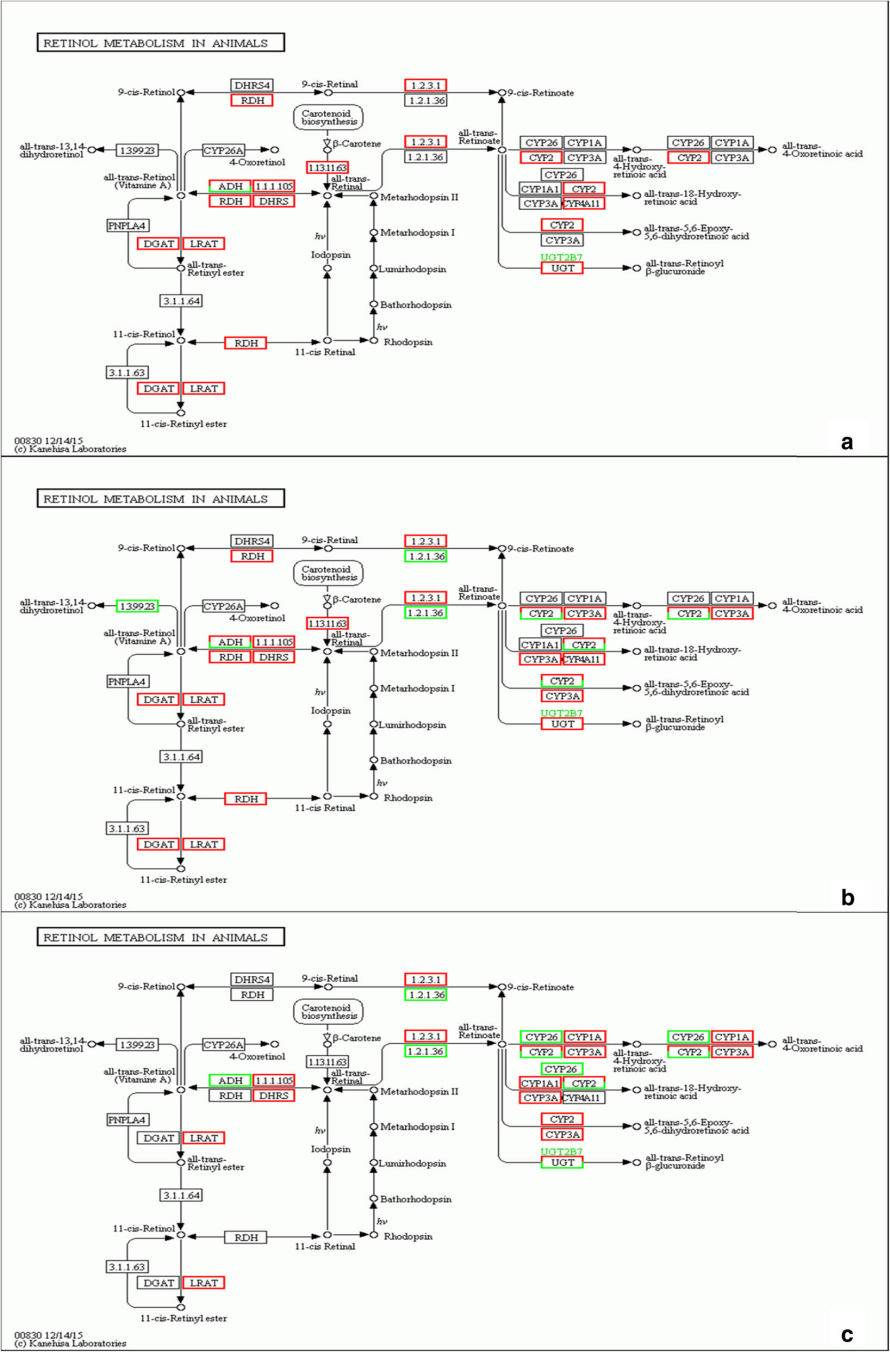
Retinol metabolism pathway changes revealed by RNA sequencing. Lesional skin compared to non-lesional skin of group A (**a**) and group B (**b**). (**c**) Differences between group A and B lesional skin. The red column indicates the genes that were upregulated, while the green column indicates the downregulated genes. With prolonged IMQ application, more molecules that are in charge of retinoic acid formation were upregulated

Focusing on retinol metabolism, we further explored the changes in expression of proteins involved downstream in the vitamin A metabolism pathway, especially those that are active after retinol is transported inside target cells. Figure 7 illustrates all of the changes detected between lesional and non-lesional skin samples as a result of RNA sequencing. At the mRNA level, enzymes that mediate retinol and RA metabolism, such as CRBP, CRABP, LRAT, RDH, and cytochromes P450 (CYP2, 3 and 4), were upregulated to different extents in lesional skin. In the two IMQ-treated groups, metabolic processes were more highly activated when the IMQ application was prolonged. Higher levels of enzymes and proteins that mediate retinol metabolic processes accumulated in lesional skin in group B than in group A, meaning that the transformation from retinol to RA was accelerated in group B (Fig. 7c).

### Serum concentrations of RBP4, STRA6, retinol, and pro-inflammatory cytokines

**Fig. 8:**
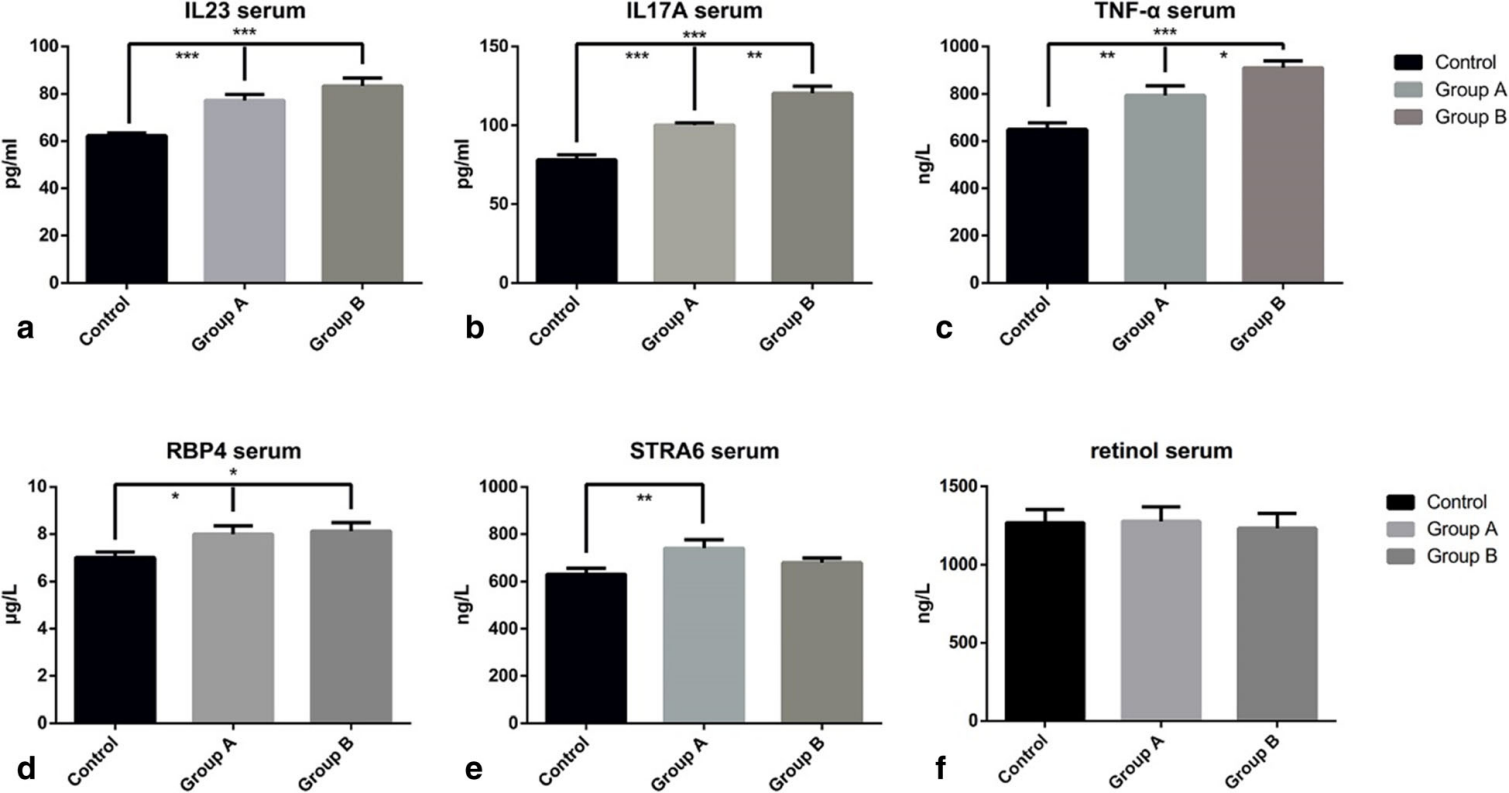
ELISA of serum changes in the study groups. Levels of IL-23 (**a**), IL-17A (**b**), and TNF-α (**c**) increased remarkably in the IMQ groups compared with controls. Between the two IMQ groups, a conspicuous increasing trend could be seen in IL-17A and TNF-α in group B. (**d**) RBP4 accumulated gradually in the circulation with the application of IMQ. Groups A and B both showed significantly increased RBP4 levels. (**e**) STRA6 levels showed a profound increase after 3 days of IMQ application; by day 7, STRA6 levels were just slightly higher than controls. (**f**) Retinol levels were slightly elevated in group A, but there were no statistically significant differences in all groups

Table 3 and Fig. 8 show the ELISA-derived serum levels of pro-inflammatory cytokines in the study groups. The levels of cytokines IL-17A, IL-23, and TNF-α were significantly higher in the IMQ groups, which could reflect the pro-inflammatory status in the psoriasis mouse model. The expression levels of these cytokines increased in a treatment duration-dependent manner (Fig. 8a-c).

The serum concentrations of target protein RBP4 were higher in groups A and B than in the controls (*p* = 0.040 and *p* = 0.021, respectively). In contrast, the 3 additional days of IMQ treatment in group B did not significantly increase RBP4 levels (*p* = 0.77), but the absolute level did increase (Fig. 8d). Because the RBP4-retinol ratio was 1:1, the concentration of apo-RBP was determined by subtracting the serum retinol concentration from the total RBP concentration. The values for group A, B and C were 5931.58 ± 294.11 ng/L, 6936.01 ± 368.04 ng/L and 6894.03 ± 436.87 ng/L, respectively (*p* = 0.029). Serum STRA6 levels were significantly higher in group A than in controls (*p* = 0.010). However, only an increasing trend existed between STRA6 levels in group B and the controls (*p* = 0.22). There was no significant difference in serum STRA6 levels between groups A and B (*p* = 0.14) (Fig. 8e). Moreover, there were no significant differences in serum retinol levels between any pair of groups. The *p* values for differences in serum retinol levels were 0.92 for group A vs. control, 0.58 for group B vs. control, and 0.59 for group A vs group B. In group A, the serum retinol level was slightly increased, but after 3 more days of IMQ application, the retinol expression decreased back to the baseline level (Fig. 8f), suggesting a dynamic change in serum vitamin A during the inception of psoriasis.

**Table 3:** ELISA of pro-inflammatory cytokines and vitamin A-related molecules in the serum of psoriasis model and control mice

Serum	Controls (*n* = 10)	Group A (*n* = 10)	Group B (*n* = 10)	*P* Value
IL-17A (pg/ml)	78.01 ± 3.31	100.34 ± 1.32	120.40 ± 4.58	<0.001***
IL-23 (pg/ml)	62.26 ± 1.16	77.31 ± 2.48	83.36 ± 3.21	<0.001***
TNF-α (ng/l)	649.25 ± 28.89	793.43 ± 41.10	909.38 ± 29.10	<0.001***
RBP4 (μg/l)	7.01 ± 0.24	8.00 ± 0.36	8.13 ± 0.36	0.042*
STRA6 (ng/ml)	631.27 ± 26.18	740.40 ± 35.62	680.18 ± 19.28	0.034*
RETINOL (ng/l)	1268.12 ± 84.95	1276.62 ± 92.91	1231.49 ± 97.07	0.793

## Discussion

We demonstrated that the effects of IMQ treatment on mice closely resembled human plaque-type psoriasis with respect to erythema, skin thickening, scaling, epidermal alterations, and inflammatory cells that infiltrated the skin and were present in the circulation, consistent with previous studies [22]. The cytokines IL-23, IL-17A, and TNF-α were elevated in both lesional skin and serum in IMQ-treated groups. With a prolonged IMQ application time, these cytokines were gradually secreted into skin lesions and serum, leading to the classic clinical phenotype. The severity of the effects of IMQ treatments appeared to build up over time.

The detection of changes in serum levels of vitamin A-related molecules in psoriasis patients dates back several decades; however, the results varied between studies. Unlike the findings of the current study, Rollman and Vahlquist found a normal mean serum RBP concentration in patients with lower PASI scores and found a significantly lower serum RBP concentration in patients with more extensive disease or pustular erythrodermic psoriasis [23]. Our results are in agreement with those of Romani et al. [14]. in that RBP4 serum levels in psoriatic subjects were significantly higher than in controls. Some studies have found that plasma RBP4 levels positively correlate with retinol levels [24]. In a study by Majewski et al., plasma vitamin A levels decreased in psoriatic patients compared with healthy controls [25]. Moreover, the more active the disease was, the lower the vitamin A levels were. Consequently, some researchers assumed that vitamin A deficiency would result in the progression of psoriasis [3, 25]. However, our results showed otherwise: no obvious changes in retinol were detected in the serum, which agreed with the findings of Demir et al. [26–28]. Furthermore, some reports indicated that serum vitamin A levels in psoriasis patients were higher than those in controls. However, this result might have been inaccurate in at least one study because the therapy factor was not taken into consideration [29]. Serum STRA6 levels in psoriasis patients have rarely been studied.

The detection of vitamin A-related molecules in psoriatic skin lesions is very limited. Many researchers proposed that retinol metabolism was altered in psoriasis lesional skin, based on the increased synthesis of RA. Rollman et al. found no differences in retinol levels in psoriatic and normal skin [23]. Many researchers have failed to detect RA levels because of technical limitations, but have proved indirectly that the synthesis of RA was upregulated [5, 23]. As a result, an assumption of increased RA levels in psoriatic skin lesions was made. In other words, additional RA is synthesized in psoriatic lesions, meaning that the demand for retinol in the skin of psoriasis patients and/or the metabolic rate of the production of RA from retinol are promoted. Our results are consistent with these assumptions.

We hypothesized that additional retinols are released from the liver to the circulation to meet the increasing demand of retinol in psoriasis lesional skin. If this is correct, more RBP4 is then needed in the circulation and in lesional skin to carry more retinol to target tissues. Additional retinol would then be transported inside target cells by the action of STRA6. In this study, proteins and enzymes such as CRBP1, LRAT, RDH, CRABP1, and CRABP2 were detected at high levels in IMQ-treated groups. If retinol stores are sufficient and the demand of retinol inside the body is low [30], retinol will be transformed into retinyl esters by LRAT and CRBP1. However, during vitamin A starvation, as in the case of psoriasis, the demand for vitamin A will increase and more retinol will interact with CRBP1 and RDH to become retinal and its functional metabolite RA. Further, interactions with CRABP1 and CRABP2 will induce RA to combine with RA receptors to effect downstream interactions, [5] such as skin proliferation and gene expression regulation. STRA6 and LRAT are considered to be RA-regulated genes [31, 32]. Consequently, STRA6 will be upregulated because of RA generation, resulting in increased retinol transport inside target cells. These processes can result in positive feedback during the pathophysiological changes associated with psoriasis.

We believe that the disease severity progressed from group A to group B, and that the effects on the vitamin A metabolism pathway became more severe. As a result of decreasing vitamin A levels, more retinol was needed and was subsequently transformed into its functional metabolites, which increased its functionality in group B. However, in the current study, retinol levels were not found with the expected expression pattern. It seems that there may be some redistribution of retinol in different tissues. Serum retinol levels are supposedly closely related to RBP4 levels, but in our experiments the RBP4 level was markedly elevated in the serum, but the retinol level was not. RBP4 has two forms in the circulation: holo-RBP4 and apo-RBP4. The holo-RBP4 is a complex in which RBP4 combines with retinol in a 1:1 ratio. After binding with STRA6 and detaching from retinol, holo-RBP4 becomes apo-RBP4 and returns to the circulation, where it encounters more retinol and once again becomes holo-RBP4. The paradox in our study could be explained by an increase in the total amounts of apo-RBP4 and holo-RBP4, wherein the amount of holo-RBP4 was about the same in both the IMQ and control groups, but more apo-RBP4 accumulated in the IMQ group serum. In our study, we used the same method that Mills et al. used to determine the levels of apo-RBP4 in different groups [33], and the result was consistent with our hypothesis. This would indicate that more retinol had already been transported into the local skin, leaving more apo-RBP4 in the circulation. Based on the redistribution theory, more retinol was likely transported into the target cells, so the detected levels of retinol in serum remained roughly the same or even slightly decreased. Meanwhile, accelerated metabolism of retinol toward RA may explain why there was no obvious elevation of retinol in IMQ lesional skin. To prove this hypothesis, the detection of RA is crucial. However, we failed to detect RA because of technical limitations.

These disparate sets of results might be attributable to many factors. In human studies, the most crucial bias is related to different recruitment criteria. Patients are diagnosed with different types of psoriasis and the disease severity varies from patient to patient. Comorbidities such as obesity, diabetes mellitus, and insulin resistance affect RBP4 levels, causing bias in detection. Another factor in these variations in research findings is probably the presence of vitamin A or its derivatives in the treatments that patients received. Therefore, a major strength of our study was the application of an IMQ-induced psoriasis mouse model to detect changes in the vitamin A metabolic pathway. This approach clearly excludes all the potential influences mentioned above. To our knowledge, this was the first study on the vitamin A metabolic pathway to include both serum and skin in psoriasis. However, our study does have some limitations. First, the detection of retinol was limited by technical considerations. A unified, simple and easy detection method will be needed to further verify our hypothesis. Second, the expression of RA and its related downstream enzymes require further study to verify the RNA sequencing results. Third, a treatment group will be needed to conversely support our results. Our attempt to build such a treatment group (using the topical vitamin A derivative tazarotene) in a previous study failed to demonstrate any improvements in psoriasis symptoms, which we attributed to local irritation and the limited experimental period. Thus another treatment model needs to be created that would help to explain why vitamin A therapy is needed when retinol levels are already elevated in psoriasis skin. Fourth, to analyze the specific functions of RBP4 and STRA6 in mouse vitamin A homeostasis, a loss-of-function study will be needed. Many researchers have chosen the STRA6 deficiency mouse as a model to study vitamin A metabolism, but they rarely pay attention to the skin. In published reports, most concluded that, except for the eye, most peripheral tissues do not display altered retinoid levels in STRA6-deficient mice [34]. A minor role of STRA6 in vitamin A uptake was considered to be consistent with the very low expression levels of STRA6 mRNA in most peripheral tissues [35]. One study revealed that STRA6 is undetectable (or is expressed at low levels) in skin, but the skin is highly responsive to retinoids [36]. This inconsistency may reflect the possibility that there are additional ways to metabolize vitamin A other than the RBP4-STRA6 pathway. Some authors have proposed that dietary vitamin A from chylomicrons could be one of these ways [35]. Skazik et al., using STRA6KD HaCaT cells, built both in vitro and in vivo experimental models that revealed that STRA6KD causes cellular vitamin A deficiency because of disturbed retinol influx, which conversely proves our theory. Consequently, STRA6 is essential for the retinoid supply to maintain retinoid homeostasis and to balance physiological regulatory effects on the proliferation and differentiation of human skin cells [8] In the serum, retinol levels were about the same in different STRA6 genotypes and vitamin A dietary groups; the same was true for RBP4 levels [37]. Thus we surmise that STRA6’s role in vitamin A metabolism is only apparent after absorption inside the target cells.

Both RBP4-deficient and RBP4 knockout models have been created in mice. Serum retinol levels in RBP4 knockout mice were reduced when compared with the wild-type controls, but there were no obvious changes in the accumulation of hepatic retinol stores [38]. These authors also discovered that the increase in serum retinol with age in RBP4 normal (RBP4^+/+^) mice is due to the increase in RBP4 mRNA expression. These results tightly connect RBP4 with retinol at the level of mRNA expression. RBP4 is a key element for retinol transportation: without it, retinol can remain in the storage form, with no function in peripheral tissues.

## Conclusion

**Fig. 9:**
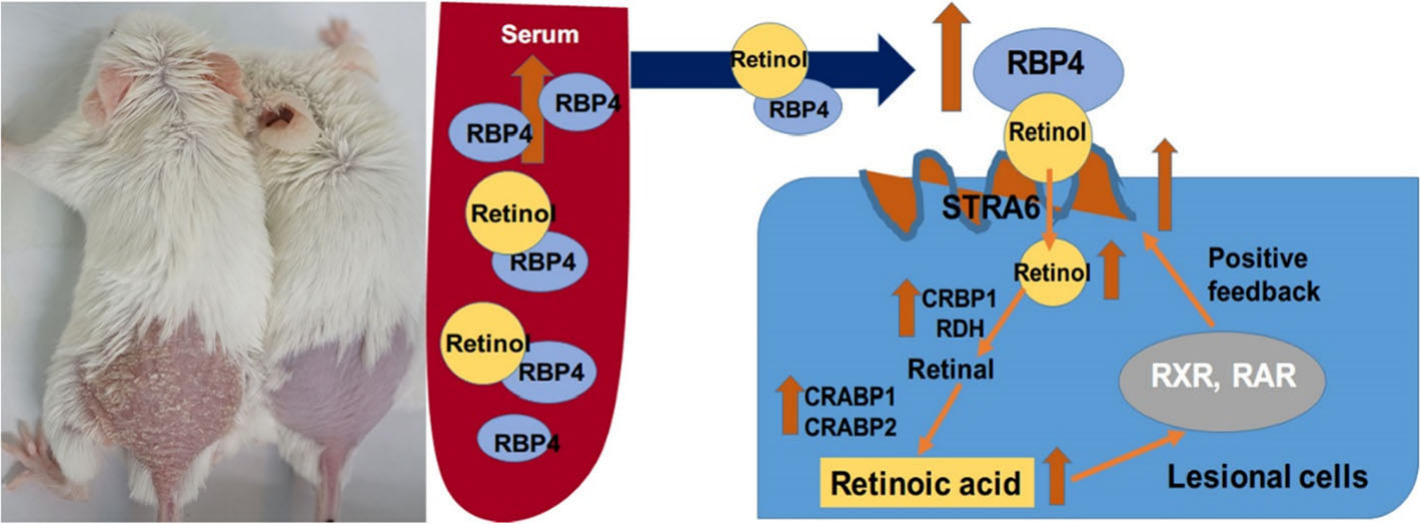
Proposed vitamin A metabolic alterations in the psoriasis model mouse. The need for retinol in psoriasis lesional skin was increased. To meet this need, RBP4 and STRA6 were upregulated in both serum and psoriatic skin to transfer more retinol inside target cells. The transformation from retinol to RA was also accelerated, by further binding with its receptor, RA induced more synthesis of STRA6, forming a positive feedback loop

The present study showed that the demand for vitamin A in psoriatic skin lesions was upregulated. In response, RBP4 and STRA6 levels increased in serum and in psoriatic skin, resulting in the transfer of more retinol to lesional skin. Retinol underwent a redistribution from the circulation to target tissues, while the transformation from retinol to RA in target tissue was accelerated, thereby inducing further synthesis of STRA6. As a result, a positive feedback mechanism was formed that maintained these psoriatic changes (Fig. 9). This might partly elucidate the mechanism of the metabolic changes associated with vitamin A in the psoriasis mouse model.

## Data Availability

The datasets used and/or analyzed during the current study are available from the corresponding author on reasonable request.
